# Suicide ideation and psychosocial distress among refugee adolescents in Bidibidi settlement in West Nile, Uganda

**DOI:** 10.1007/s44202-021-00003-5

**Published:** 2021-10-04

**Authors:** Paul Bukuluki, Peter Kisaakye, Symon Peter Wandiembe, Samuel Besigwa

**Affiliations:** 1 Department of Social Work and Social Administration, School of Social Sciences, Makerere University, Kampala, Uganda; 2 Department of Population Studies, School of Statistics and Planning, Mathematics Building, Makerere University Main Campus, P O Box 7062, Kampala, Uganda; 3 Department of Statistical Methods, School of Statistics and Planning, Makerere University, Kampala, Uganda

**Keywords:** Adolescents, Bidibidi, Psychological distress, Refugees, Suicide attempt, Suicide ideation, Uganda

## Abstract

This study investigated the factors associated with suicidal ideation and psychological distress among Sudanese refugees (aged 10–19 years) in Bidibidi refugee settlement, Yumbe district, Uganda. The analysis is based on cross-sectional data collected as part of the implementation of a project on sexual and reproductive health in Bidibidi refugee settlement. Data were collected from 284 South Sudanese adolescents in July 2020. We estimated the prevalence of psychological distress and suicidal ideation, and their associated profiles. Odds ratios and their 95% Confidence Intervals based on a logistic regression model were used to assess the effect of different potential factors on the risk profiles for suicidal ideation and psychological distress. The prevalence of psychological distress (on KS-6 scale) was estimated to be 12.3% (95%CI: 8.7, 16.7%). The risk for psychological distress is low among male adolescents (AOR = 0.51; 95%CI: 0.23, 1.02), older adolescents, (AOR = 0.12; 95%CI: 0.04, 0.40), and adolescents with a friend or family members they can confide in (AOR = 0.33; 95%CI: 0.11, 0.96). Both suicidal ideation and psychological distress are very high among the female adolescents. Familial and individual interventions can be potentially useful for female adolescents who have a high risk of suicide and psychological distress.

## Introduction

Suicide ideation (SI) or suicide attempt (SA) remain a public health concern [1]. The World Health Organization (WHO) estimates about 800,000 deaths from suicide related causes annually [2]. Suicide has also been reported as a leading cause of death among adolescents and youth living in refugee settings, particularly in the age group 15–29 years [1, 3]. Adolescents are generally most vulnerable to suicide as this stage involves highly continuous mental and physical developments [4, 5]. Despite the alarming situation about suicidality, there is limited knowledge on the risk of suicide behaviors among vulnerable populations such as adolescent refugees [1, 3, 6, 7]. Moreover, there is limited literature on SI and SA among vulnerable populations such as refugee adolescents [1, 3].

Data from middle-school immigrant adolescents from North-Eastern France revealed a two-time higher risk of experiencing SI and SA than non-migrants [3]. This implies that educated people may be less likely to experience SI and SA because of an increase in social mobility [8]. A recent study consisting of 251 emerging adults from two universities in South-West Nigeria revealed that an increase in family support leads to a decrease in SI and SA due to a decrease in depression [1]. According to Olatunji and colleagues, family support is part of social support that helps in providing material, financial and emotional support [1].

Previous research estimated suicide ideation to be 21% in a rural Ugandan population [9]. Others estimated suicide ideation and suicide attempt to be 12 and 6% among respondents aged 18 and above in Gulu, Amuru and Nwoya districts in Uganda [7]. A population-based study conducted in post-conflict Northern Uganda revealed that suicidality was more prevalent among individuals with a depression disorder and females [7].

The literature points to several factors associated with suicide ideation (SI) or suicide attempt (SA). These include feeling or being lonely [10], discrimination [11], financial constraints [3], anger [12], mental illness [7, 13], political tyranny [5], depression [14, 15], alcohol abuse [16], substance abuse [17, 18], unemployment [19], stress or posttraumatic stress disorder [20, 21], acculturation related stress [6], war [22], trauma [23], isolation [24], family instability or conflicts [12, 25], history of suicide behaviours [26], violence [27], or gender-based violence [28, 29], sexual abuse [30], child abuse or bullying [31], and not having a close friend [32].

There is need to identify the risk factors associated with SI and SA among vulnerable populations such as refugee adolescents since they are of different education, behavioral, social, health, employment and substance use factors. Suicide ideation or attempt behaviors among refugee adolescents remain a complex dimension to understand [3]. Understanding the risk factors associated with SI and SA may help in devising prevention strategies against experiencing SI and SA. Previous research [33, 34] has observed that refugee adolescents are more likely to have parents or guardians with low education, poor health and socioeconomic background as well as disjointed families which are probable predictors of SI and SA [35].

The aim of this study was to investigate the prevalence and risk factors of suicide ideation (SI) and psychosocial distress among adolescents in Bidibidi refugee settlement in Uganda. We focus on adolescent refugees who are mostly immigrants because previous studies have reported a higher rate of suicide ideation and attempt among immigrants than native populations [3, 5, 6, 14]—calling for selective preventive strategies to assist vulnerable groups needed [3]. This study was conducted during the COVID-19 pandemic when there were restrictions on movements, which could have affected living conditions of people. For example, refugees in Uganda are allowed to move in and out of their refugee camps to engage in commercial and other activities [36, 37]. However, travel restrictions, could have had a negative impact on the welfare of refugees especially for those who could not move either to meet their families or engage in any commercial and social activities outside refugee settlements [38].

### Theoretical framework

This paper draws on the interpersonal theory of suicidal behaviour (IPTS) [39]. The IPTS is one of the most theories used to understand suicide behaviour [40]. The theory posits that two conditions (proximal factors) must be present for suicide ideation to happen: perceived burdensomeness and thwarted belongingness). According to the theory, people get suicidal ideations when their perceived level of burdens (perceived burdensomeness) and feeling lonely increases thwarted belongingness). Perceived burdensomeness refers to when someone thinks their existence is a burden to people around them such as family members or friends. Thwarted belongingness refers to when thinks they do not belong to any group or family or friends [41, 42]. As suicidal ideations increase, people tend to carry out suicide attempts [43]. The implication of this is that suicide attempt can only happen after suicide ideation. Joiner and colleagues argue that people attempt suicide after positioning themselves in a state where they have overcome the fear of injury, death or even pain [44]. Drawing on the life course theory, suicide attempts may also be caused by other environmental or social factors. For example, the risk of committing a suicide among adolescents may increase due to death of a parent, family conflict, limited parental support which may limit progress to adult life [45–47].

Based on the literature, we hypothesize that adolescents who have a friend or family member to confide in were less likely to experience suicide ideation or psychosocial distress. Adolescents who are aware of or have ever accessed psychosocial support were less likely to experience suicide ideation or psychosocial distress.

## Data and methods

This study investigated the factors associated with suicidal ideation and psychological distress among 281 Sudanese refugees (aged 10–19 years) in Bidibidi refugee settlement, Yumbe district in Uganda. The site was chosen because it is the largest refugee settlement in Uganda. It hosts largely South Sudanese refugees, primarily from the Equatorial region. The analysis is based on data collected during a cross-sectional study on adolescent sexual, reproductive health and psychosocial wellbeing. This study was conducted by Makerere University using a team of trained researchers based in Yumbe district where the Bidibidi refugee settlement is located. Given that it was during the COVID-19 pandemic, the research team had to strictly observe all COVID-19 Standard Operating procedures (ensuring wearing of masks for both the interviewers and respondents, sanitizing and observing social distancing) as recommended by the World Health Organization [48] to ensure no harm to the participants and the research team. A structured questionnaire was developed based on the study objectives, and data were collected using the Computer Assisted Personal Interviewing (CAPI) technology.

A probability-based sample approach was employed to select 300 eligible adolescents for interviews. The computation of the sample size to assure estimation of adolescents was based on the sample size tables by Krejcie and Morgan [49]. From on literature [7], we assumed suicidal attempt among adolescents to be 10%. A sample size of 300 adolescents was sufficient to assure estimation of this proportion with 25% relative standard error and 95% certainty, while allowing for non-response rate of 5% and cluster design effect of 2.0.

A two-stage cluster sample was taken. In the first stage, a list of all blocks (a block is an equivalent to a village) in the settlement and their sizes (number of households) were obtained from the settlement commandant’s office. A probability proportional to size sample of 30 blocks was taken. Within each selected block, the research team worked with Refugee Welfare Council (RWC) who facilitated the process of identifying households with at least one adolescent (10–19 years). A numbered list of households with adolescents within each sampled block was generated and a systematic sample of ten taken. The total of eligible households, N, divided by 10 was used as a sampling interval in each block. The selection of the first household was automated in the CAPI. Within each sampled household, a list of all adolescents was generated and numbered. A lottery method was used to select one adolescent for the interview.

Out of the estimated total sample size of 300 adolescents (10–19 years), only 284 adolescents responded to the interview—giving a response rate of 95%. Permission to conduct the study was granted by the School of Social Sciences Research Ethics Committee at Makerere University. Each respondent was interviewed separately, with permission from the participant and their parent or guardian(s) (consent and assent) sought through the consenting process facilitated by an informed consent form. The interview duration ranged from 45 min to 1 h.

The depressive symptoms were assessed using KS-6 scale, a well-studied and validated scale used for adolescents (Kessler et al. 2002; Richwood et al. 2015). The score sum ranged from 0 to 24. Similar to the study by Richwood et al. 2015, a cutoff point of 10 was used to differentiate depressed versus non-depressed individuals. Cronbach’s alpha reliability coefficient in our sample was estimated as 0.78.

Variables that were used in our analysis included socio-demographic characteristics: age (10–12, 13–15, 16–19), education (primary, secondary), religion (Anglican, Catholic, Other), employment status (employed, not employed, student), HIV/AIDS (negative, positive, do not know), alcohol abuse (no, yes), drug and substance abuse (no, yes), have a friend or family member you can confide in (no, yes), aware of places for psychosocial support (no, yes) and ever accessed psychosocial support (no, yes). The questions on suicidality and psychosocial support included: (a) Have you ever had thoughts of killing yourself in the last 4 weeks?, (b) Have you ever had thoughts of killing yourself in the last one week?, (c) Have you tried killing yourself in the last six months? (d) Do you have hope for the future?, and experience of psychosocial distress. Responses to each of the questions were a yes or no.

Data analysis was performed using Stata software version 15.0 (Stata Corporation, College Station, TX, USA). Descriptive statistics including percentages and frequencies were used to summarize categorical variables. A binary logistic regression model was used to assess the factors associated with suicide ideation and psychosocial distress.. The results are reported as proportions, with odds ratios (OR) and their 95% confidence intervals (95%CI). Only factors with log likelihood ratio test p-value of at most 0.25 in the first model were included in the second model.

## Results

### Characteristics of respondents

Of the 281 adolescents that participated in the study, 69% were male, 12.5% were aged below 12 years and 53% were aged 13–15 years (Table 1). Ninety four percent (94.2%) had only primary school level education and 51.2% (74% of the girls and 41% of the boys) were currently schooling. All the adolescents except three girls were married or cohabiting. About 47% reported to have ever tested for HIV, of which only one self-reported to be HIV positive. Only 1.4% reported alcohol abuse and 1.4% had ever used illicit substances. Of those out of school, only 5% were employed. Over 90% reported to have friends in whom they could confide in about relationship and psychosocial issues, 33.5% were aware of a place to seek psychosocial support and counselling and overall, 32.4% had ever sought psychosocial support and counselling services.

**Table 1:** Sample characteristics

	Female adolescents		Male adolescents		Total	
	n	%	n	%	n	%
Age group						
Very younger adolescents (10–12 years	19	22.1	16	8.2	35	12.5
Younger adolescents (13–15 years old)	44	51.2	105	53.8	149	53.0
Older adolescents (16–19 years old)	23	26.7	74	37.9	97	34.5
Education level						
Primary	75	89.5	191	98.5	266	94.2
Secondary	11	14.0	4	2.1	15	5.4
Religious affiliation						
Anglican	34	39.5	103	52.8	137	48.8
Catholic	32	37.2	63	32.3	95	33.8
Other	20	23.3	28	14.4	48	17.1
HIV/AIDS status (Self-reported)						
I do not know	53	61.6	96	49.2	149	53.0
Negative	33	38.4	98	50.3	131	46.6
Positive	0	0.0	1	0.5	1	0.4
Alcohol Abuse						
No	83	96.5	193	99.0	276	98.2
Yes	3	3.5	1	0.5	4	1.4
Drug and substance abuse						
No	85	98.8	191	97.9	276	98.2
Yes	0	0.0	4	2.1	4	1.4
Employment status						
Employed	0	0.0	6	3.1	6	2.1
Not Employed	22	25.6	108	55.4	130	46.3
Student	64	74.4	80	41.0	144	51.2
Have a friend/family member s/he can						
No	7	8.1	18	9.2	25	8.9
Yes	78	90.7	177	90.8	255	90.7
Aware of places for psychosocial support						
No	53	61.6	133	68.2	186	66.2
Yes	32	37.2	62	31.8	94	33.5
Ever accessed psychosocial support						
No	54	62.8	129	66.2	183	65.1
Yes	31	36.0	60	30.8	91	32.4
Prevalence of suicide ideation and Psychological distress						
Suicide ideation in last 4 weeks	10	11.6	5	2.6	15	5.3
Suicide ideation in last one week	7	8.5	1	0.5	8	3.0
Suicide ever attempted	3	3.5	1	0.5	4	1.4
Suicide attempt in last six months	1	1.2	1	0.5	2	0.7
Do not have hope for the future	12	14.0	21	10.8	33	11.7
Psychological distress	16	18.6	18	9.2	34	12.1

### Prevalence of suicide ideation and psychological distress

The prevalence of suicidal ideation in the past four weeks was estimated as 5.3% (95% CI: 3.1, 7.4%) while suicidal attempt in the last six months preceding the study was reported by 0.7% (95% CI: 0.3, 1.2%) of the adolescents. The prevalence of both ideation and attempt among female adolescents was more than twice the prevalence among the female adolescents. Prevalence of suicidal ideation was 12.6% among girls and 3.6% among the boys while a suicide attempt was 3.5% and 0.9% among the female and adolescents, respectively. The prevalence of psychological distress (on KS-6 scale) was estimated to be 12.1% (95%CI: 8.7, 16.7%). This was dominated by lack of hope in the future, estimated at 11.7% (95%CI: 8.1, 15.9%). Similar to suicide ideation, more female than male adolescents reported psychological distress (18.6 vs. 9.2%).

### Factors associated with suicidal ideation or psychological distress

At univariate analysis (Table 2), male adolescents (OR 0.20; 95%CI: 0.07, 0.60) or having a friend/ family member/ relative to confide in was associated with (OR 0.16; 95%CI: 0.05, 0.50) had less experience with suicide ideation in the last four weeks. On the other hand, males (OR 0.44; 95%CI: 0.21, 0.92) were less likely to be psychologically distressed compared to females. Adolescents in the aged 13–15 years (OR 0.11; 95%CI: 0.04, 0.29) and those aged 16–19 years (OR = 0.35; 95%CI: 0.14, 0.84) were all less likely to be associated with psychological distress compared to adolescents aged 10–12 years. Adolescents with some secondary school education (OR 3.98; 95%CI: 1.28, 12.33) were more likely to experience psychological distress than adolescents with primary education only. Similarly, adolescents who reported that they do not have hope in the future (OR 29.63; 95%CI: 12.16, 72.20) were more likely to experience psychological distress than their counterparts.

**Table 2:** Univariate analysis: Multivariate analysis – logistic regression model of factors associated with suicide ideation or Psychological distress

	N	Suicide ideation in last 4 weeks			Psychological distress		
		n	%	OR (95%CI)	n	%	OR (95%CI)
Sex							
Female adolescents	86	10	11.6	Ref	16	18.6	Ref
Male adolescents	195	5	2.6	0.2 (0.07, 0.6)*	18	9.2	0.44 (0.21, 0.92)*
Age group							
Very younger adolescents (10–12 years)	35	2	5.7	Ref	12	34.3	Ref
Younger adolescents (13–15 years)	151	7	4.6	0.8 (0.16, 4.04)	8	5.3	0.11 (0.04, 0.29)*
Older adolescents (16–19 years)	98	6	6.1	1.08 (0.21, 5.60)	15	15.3	0.35 (0.14, 0.84)*
Education level							
Primary	269	15	5.6	Ref	30	11.2	Ref
Secondary	15	0	0		5	33.3	3.98 (1.28, 12.33)*
Religious affiliation							
Anglican	138	3	2.2	Ref	15	10.9	Ref
Catholic	96	3	3.1	1.45 (0.29, 7.35)	10	10.4	0.95 (0.41, 2.22)
Other	50	9	18	9.88 (2.55, 38.2)	10	20	2.05 (0.85, 4.92)
Have a friend/family member s/he can confide in							
No	25	5	20	Ref	6	24.0	Ref
Yes	258	10	3.9	0.16 (0.05, 0.5)*	28	10.9	0.39 (0.14, 1.05)
Aware of places for psychosocial support services							
No	188	12	6.4	Ref	25	13.3	Ref
Yes	95	3	3.2	0.48 (0.13, 1.74)	10	10.5	0.77 (0.35, 1.67)
Ever accessed psychosocial support services							
No	185	11	5.9	Ref	23	12.4	Ref
Yes	92	3	3.3	0.53 (0.15, 1.96)	9	9.8	0.76 (0.34, 1.73)
Do not have hope in the future							
No	251	12	4.8	Ref	14	5.6	
Yes	33	3	9.1	2.00 (0.53–7.43)	21	63.6	29.63 (12.16, 72.2)*
Total	284	15	5.3		35	12.3	

Multivariable analysis based on a logistic regression model is presented in Table 3. At multivariate analysis, the risk for suicide ideation was low among male adolescents (AOR = 0.16; 95%CI: 0.05, 0.52) and adolescents with a friend or family members they can confide in (AOR = 0.14; 95%CI: 0.04, 0.50). Similarly, the risk for psychological distress is low among male adolescents (AOR = 0.51; 95%CI: 0.23, 1.02), older adolescents as compared to those aged 10–12 years (AOR = 0.12; 95%CI: 0.04, 0.40), and adolescents with a friend or family members they can confide in (AOR = 0.33; 95%CI: 0.11, 0.96).

**Table 3:** Multivariate analysis – logistic regression model of factors associated with suicide ideation or Psychological distress

	N	Suicide ideation in last 4 weeks		Psychological distress	
		Adjusted OR (95%CI)	p-value	Adjusted OR (95%CI)	p-value
Sex					
Female adolescents	86				
Male adolescents	195	0.16 (0.05, 0.52)	0.002	0.38 (0.14, 1.05)	0.061
Age group					
Very younger adolescents (10–12 years)	35				
Younger adolescents (13–15 years)	151	1.17 (0.19, 7.12)	0.862	0.23 (0.05, 1.03)	0.05
Older adolescents (16–19 years)	98	1.95 (0.31, 12.41)	0.477	0.68 (0.16, 2.86)	0.60
Education level					
Primary	269				
Secondary	15			0.44 (0.06, 3.38)	0.43
Have a friend/family member s/he can confide in					
No	25				
Yes	258	0.14 (0.04, 0.5)	0.003	0.46 (0.12, 1.72)	0.25
Do not have hope in the future					
No	251				
Yes	33	1.32 (0.28, 6.27)	0.729	29.5 (10.81, 50.56)	0.00

## Discussion

The results indicate a higher prevalence of suicide ideation and psychological distress among female than male refugee adolescents. Our findings are similar to those of Culbreth and colleagues who found a high occurrence of suicide ideation among female youth in Uganda [50]. For the Ugandan refugee context, this could among other factors be attributed to the prevalence of other risk factors that affect female adolescents more than the males such as gender based violence [28, 29]. Girls are for example likely to be subjected to limited mobility (control of mobility), sexual abuse and exploitation as well as burden of care reflected in taking on more domestic chores [30]. These are likely to affect the extent to which they can build social networks and have access to activities outside their immediate social environment. Our study also shows that adolescents with a friend or family member to confide in were less likely to experience suicide ideation in the last four weeks preceding the survey. This implies that having a limited social network of friends, relatives and contact with family [50] increases loneliness and isolation. Other studies also have also established that suicide ideation and suicide attempt is associated with feeling or being lonely [10], isolation [24], and not having a close friend [32]. In our study, refugee adolescents who reported that they do not have hope in the future were more likely to experience psychological distress. It is important to note that because this study was conducted during the COVID-19 pandemic context, it may have exacerbated the loss of hope for the future and loss of contact with friends and family. For example, adolescents were no longer attending school, and this limits their interaction with their peers in the host population and at school. Similarly, COVID-19 control measures contributed to limited mobility for family members and friends who do not live in the refugee settlement but in cities in Uganda as urban refugees to visit their families and friends who live in refugee settlements. In Uganda, before COVID-19 restrictions were imposed, refugees were generally allowed to move in and outside the refugee camps and to even move to urban cities to work and engage in trade [36, 37]. So those who moved to urban areas for a number of months especially during the lockdown and stay home orders could not easily travel back to join their family members living in the refugee camps [38].

Loss of hope for the future among adolescent refugees tends to be linked to low disjointed families [33, 34] that comprise largely of women and children (World Bank, 2019). A household survey conducted by the World Bank in 2018 in refugee settlements in Uganda revealed that the women and children comprise about 82 percent of Uganda’s overall refugee population while about 56 percent of refugees are below the age of 15 [35]. Families with adolescents in refugee settlements are also likely to have parents with low education, poor health and socioeconomic background which are likely to render adolescents susceptible to SI and SA [33].

Our results also show limited awareness where to access psychosocial support services among refugee adolescents and no gender differences were observed. They also show limited access and use of psychosocial support services. This is similar to results of a recent World Bank study in 11 refugee settlement (including Bidibidi settlement) that found that availability and access to mental health and psychosocial support services is low in refugee settlements in Uganda [35].

Adolescents with secondary education were more likely to experience psychological distress than adolescents with primary education. This could be explained by the fact that adolescents with secondary education tend to have higher expectations and if these are not met, they are likely to feel frustrated and unhappy compared to their counterparts with lower levels of education. Education has been shown to increase expectations for social mobility [8] and if these are not me it may increase risks of psychological distress.

## Conclusion

Overall refugee adolescents, especially female adolescents are susceptible to suicidal ideations and attempts. The major risk factors associated with suicidal ideations and attempts include loneliness, isolation, not having friend or family member to confide in, having no hope for the future and limited or no access to mental health and psychosocial support services. Given these risk factors, it is important to ensure that mental health and psychosocial support is integrated in interventions and services targeting adolescents. These services can be integrated in school, out of school and in health services provided to adolescents. There is need to strengthen surveillance of suicidal ideations and attempts among all refugees particularly female adolescents. This more so, during global pandemic like COVID-19 that entail using measures that may further contribute to isolation, loneliness and loss of social contact, confinement at home within the camps and reduced mobility for adolescent refugees. We also recommend studies with a larger sample size to be carried out to further galvanize efforts geared towards generating evidence for advocacy for mental health and psychosocial services targeting adolescent refugees.

## Limitations

There are three main limitations we note in this study. First, we are unable to generalize our results to the entire adolescent refugee populations because the results generated from the analyses in this study are based on a relatively smaller sample. Second, this study was conducted during the COVID-19 pandemic. For this reason, we were unable to incorporate qualitative and interactive interviews in our study, and last, the results we report in this study could have been affected by the effect of COVID-19 pandemic, but we were not able to control for that.

## Data Availability

The datasets used in this study are available from the corresponding author on reasonable request.

## Abbreviations

CAPI: Computer Assisted Personal Interviewing
OPM: Office of the Prime Minister
RWC: Refugee Welfare Council
SA: Suicide Attempt(s)
SI: Suicide Ideation(s)
UNHCR: United Nations High Commissioner for Refugees
WHO: World Health Organisation
